# "Abolishing" the Slums

**Published:** 1923-12

**Authors:** 


### "ABOLISHING" THE SLUMS.

IN its General Election Manifesto the Labour Party declares that, if it comes into power, it will "abolish slums." With so fine an aspiration every voter will sympathise; but the "abolition" of slums is only a shade less difficult than the "abolition" of poverty. It is impossible, by waving a magic wand, to rehouse great numbers of people, and a Labour Government would find itself faced by the same practical difficulties that have hitherto hampered and, in great measure, must continue to hamper, both central and local administrations. Slum clearance is a costly process, and comparatively little can be done in that direction until homes have been found for the dispossessed. To demolish a rookery before providing other accommodation would be folly, even if it were practicable and legal; demolition is only one side of a scheme the other side of which consists of rehousing. It is idle for one political party to blame another for a situation which is universally deplored and is of old standing. It has been produced by past neglect and indifference, and has of late years been sharpened by economic disturbances resulting from the war. It must not be forgotten that the slum question was already in active existence when there was a surplus, instead of a scarcity, of small houses. The scarcity has merely exacerbated it.

The whole subject is inextricably mixed up with town-planning, as we are reminded by the admirable paper read recently to the British Association by Mr. John J. Clarke, which has just been published by that University of Liverpool Press which is helping to make known so many admirable schemes of urban and regional improvement. No town of any size is free from slums, and it does not necessarily follow that the town-planning projects which every community of more than 20,000 people is now bound to prepare will get rid of them. When these projects acquire legal force they should prevent, or go far to prevent, the creation of new rookeries. That will be a very important gain, and very often new and airy thoroughfares will be driven through districts which are insanitary, both physically and morally. But when we come to deal with hundreds, or thousands, or even, in some cases, tens of thousands of back-to-back dwellings, the problem calls for the exercise of practical statesmanship of the first order. And, be it observed, it must, in the first instance, be municipal statesmanship. No central authority can prepare and impose detailed schemes for cities such as Birmingham, Liverpool and Sheffield. The opportunity for the development of high municipal policy and finance is wider than it has ever been, and in the great towns there is as much need for leadership as in national affairs.

In the paper to which we have referred, Mr. Clarke insists upon the need of organising the nation for a war upon slums. He would recruit the younger men who are unemployed, and have little present chance of obtaining employment, and form them into Housing Battalions of the Royal Engineers and, after training them, set them to work to build houses. There is something in an idea which would give the nation a solid return for money which is at present in great measure wasted in the shape of its contribution to the maintenance of men who are out of work. We fear, however, that the supply of really skilled men, such as bricklayers and plasterers, would fall so short that the scheme could not be matured rapidly. Moreover, are we likely to have a Government that would brave the wrath of the Trade Unions? Modern Governments are so terrified of these great organised interests that we do not believe one could be found to do it, even on the ground of national emergency. For the time being, therefore, we can only hope that the next eighteen months' experience of the new Housing Act will be sufficiently satisfying to admit of a national anti-slum campaign being prepared in readiness for the time when rehousing on a considerable scale becomes possible. An evil that has been growing for a century or more cannot be eradicated quickly. Yet health, morality and care for the dormant self-respect of the slum-dweller demand that we should not lose time in removing a peculiarly bad blot upon our reputation as a people.

